# Supplementary material for: Oral history as a citizen science tool to understand biodiversity loss and environmental changes: on firefly extirpation in Morelia, Michoacán, Mexico
Source: PeerJ. 2025 May 5;13:e19413. doi: 10.7717/peerj.19413 (PMC12060901; doi:10.7717/peerj.19413)
Supplement: Supplemental Information 3 — Anecdotal experiences and testimonies of local extirpation of firefly populations at these sites were previously recorded. The sites were physically surveyed in 2022 and 2023 by firefly specialists of the Project “Luciérnagas de Michoacán” to record the current absence of nocturnal fireflies. [file peerj-13-19413-s003.docx]

Supplementary Materials. Rivera-Ramírez et al. Oral history as a citizen science tool to understand biodiversity loss and environmental changes: a case study on firefly extirpation in Morelia, Michoacán, Mexico.

**Table S1. Firefly extirpation sites reported by the middle and oldest generations of Morelian**. Anecdotal experiences and testimonies of local extirpation of firefly populations at these sites were previously recorded. The sites were physically surveyed in 2022 and 2023 by firefly specialists of the Project “Luciérnagas de Michoacán” to record the current absence of nocturnal fireflies.

| **Species** | **Lat, Long** | **Compiler** | **Reporting year** | **Citation or source** |
| --- | --- | --- | --- | --- |
| Lampyridae sp | 19.6683°N, -101.2262°O | Rivera-Ramírez, D.B. | 2022 | Pérez-Hernández CX, Mendoza-Cuenca LF, Romo-Galicia A. 2023b. Dataset of the Lampyridae (Coleoptera) from Morelia, Michoacán, México. Universidad Michoacana de San Nicolás de Hidalgo Facultad de Biología. Available at https://doi.org/10.15468/3asucg |
| Lampyridae sp | 19.6858°N, -101.1995°O | Rivera-Ramírez, D.B. | 2022 | Pérez-Hernández CX, Mendoza-Cuenca LF, Romo-Galicia A. 2023b. Dataset of the Lampyridae (Coleoptera) from Morelia, Michoacán, México. Universidad Michoacana de San Nicolás de Hidalgo Facultad de Biología. Available at https://doi.org/10.15468/3asucg |
| Lampyridae sp | 19.6917°N, -101.1855°O | Rivera-Ramírez, D.B. | 2022 | Pérez-Hernández CX, Mendoza-Cuenca LF, Romo-Galicia A. 2023b. Dataset of the Lampyridae (Coleoptera) from Morelia, Michoacán, México. Universidad Michoacana de San Nicolás de Hidalgo Facultad de Biología. Available at https://doi.org/10.15468/3asucg |
| Lampyridae sp | 19.6933°N, -101.2054°O | Rivera-Ramírez, D.B. | 2022 | Pérez-Hernández CX, Mendoza-Cuenca LF, Romo-Galicia A. 2023b. Dataset of the Lampyridae (Coleoptera) from Morelia, Michoacán, México. Universidad Michoacana de San Nicolás de Hidalgo Facultad de Biología. Available at https://doi.org/10.15468/3asucg |
| Lampyridae sp | -101.2073°O | Rivera-Ramírez, D.B. | 2022 | Pérez-Hernández CX, Mendoza-Cuenca LF, Romo-Galicia A. 2023b. Dataset of the Lampyridae (Coleoptera) from Morelia, Michoacán, México. Universidad Michoacana de San Nicolás de Hidalgo Facultad de Biología. Available at https://doi.org/10.15468/3asucg |
| Lampyridae sp | 19.7029°N, -101.1913°O | Pérez-Hernández, C.X. | 2023 | Stand of the “Luciérnagas de Michoacán” Project during the “Tianguis de la Ciencia” 2023 |
| Lampyridae sp | 19.6913°N, -101.1807°O | Pérez-Hernández, C.X. | 2023 | Stand of the “Luciérnagas de Michoacán” Project during the “Tianguis de la Ciencia” 2023 |
| Lampyridae sp | 19.6964°N, -101.1828°O | Pérez-Hernández, C.X. | 2023 | Stand of the “Luciérnagas de Michoacán” Project during the “Tianguis de la Ciencia” 2023 |
| Lampyridae sp | 19.6872°N, -101.1912°O | Pérez-Hernández, C.X. | 2023 | Stand of the “Luciérnagas de Michoacán” Project during the “Tianguis de la Ciencia” 2023 |
| Lampyridae sp | 19.6625°N, -101.2265°O | Pérez-Hernández, C.X. | 2023 | Stand of the “Luciérnagas de Michoacán” Project during the “Tianguis de la Ciencia” 2023 |
